# Bridging the care continuum: patient information needs for specialist referrals

**DOI:** 10.1186/1472-6963-9-163

**Published:** 2009-09-15

**Authors:** Carol L Ireson, Svetla Slavova, Carol L Steltenkamp, F Douglas Scutchfield

**Affiliations:** 1 College of Public Health, University of Kentucky, Lexington, Kentucky, USA; 2 Office of Institutional Research, University of Kentucky, Lexington, Kentucky, USA; 3 College of Medicine, University of Kentucky, Lexington, Kentucky, USA; 4 College of Public Health, University of Kentucky, Lexington, Kentucky, USA

## Abstract

**Background:**

Information transfer is critical in the primary care to specialist referral process and has been examined extensively in the US and other countries, yet there has been little attention to the patient's perspective of the information transfer process. This cross-sectional study examined the quality of the information received by patients with a chronic condition from the referring and specialist physician in the specialist referral process and the relationship of the quality of information received to trust in the physicians.

**Methods:**

Structured telephone interviews were conducted with a random sample of 250 patients who had experienced a referral to a specialist for the first visit for a chronic condition within the prior six months. The sample was selected from the patients who visited specialist physicians at any of the 500 hospitals from the National Research Corporation client base.

**Results:**

Most patients (85%) received a good explanation about the reason for the specialist visit from the referring physician yet 26% felt unprepared about what to expect. Trust in the referring physician was highly associated with the preparatory information patients received. Specialists gave good explanations about diagnosis and treatment, but 26% of patients got no information about follow-up. Trust in the specialist correlated highly with good explanations of diagnosis, treatment, and self-management.

**Conclusion:**

Preparatory information from referring physicians influences the quality of the referral process, the subsequent coordination of care, and trust in the physician. Changes in the health care system can improve the information transfer process and improve coordination of care for patients.

## Background

The experience of being referred to a specialist provider is a significant issue for patients in the United States health care system. In this age of increasing specialized knowledge about chronic diseases, patients receive care from multiple physicians across a variety of settings. According to the 2006 National Ambulatory Medical Care Survey, patients made nearly 902 million visits to physician and over 46% (421 million) of those visits were to a specialist provider. Of the specialist provider visits, 137 million (33%) resulted from a referral from another provider [1]. A specialist referral requires coordination and integration of care, and transition and continuity of care- two of the seven dimensions of patient-centered care [2].

Emanuel and others developed a prototype of the relationship that should underpin the tripartite relationship in a consultation experience, involving the patient, the primary care physician (PCP) and the specialist physician (SP) [3,4]. Williams et al suggested that the referral process involved three steps requiring coordination: (1) the referring physician communicates the reason for the referral and pertinent patient information to the specialist, (2) the specialist completes the referral and communicates findings to the referring physician, and (3) the referring physician, specialist and patient negotiate continuing care arrangements [5]. Coordination of care has been narrowly defined as the information exchange among care providers to ensure that care is directed to a common goal [6]. Most of the research about specialty referrals to date has focused on information exchange between health care providers. These studies show that primary care to specialists referrals lack sufficient information exchange [7-9].

Coordination of care also takes places between providers and patients and families [10]. Information exchange between providers and patients also has been found to be lacking. In one study, patients reported receiving conflicting advice from different physicians that according to the investigators was likely because of communication breakdown between providers [11]. A survey in 48 Massachusetts hospitals revealed patient dissatisfaction with continuity and transitions, information and education, and coordination of care [12]. A recent study found that nearly one third of physicians did not notify patients of abnormal diagnostic test results [13].

Studies of specialist referrals reveal that providers failed to discuss a referral with 27% of patients who indicated a definite desire for a referral and 56% of patients communicating a possible desire [14]. In a subsequent intervention study, a pre-visit questionnaire increased provider awareness and improved patient satisfaction with the visit [15]. The referring physician plays a critical role in facilitating the patient's completion of a referring by assisting in the scheduling of the referral [16].

Referring physicians are critical to the provision of preparatory information for the specialist visit. Likewise, the information that the specialist subsequently gives the patient about diagnosis, treatment, and follow-up is not only important immediately to the patient, but also for the coordination of the patient's care with the primary care provider. Getting understandable information increases patient satisfaction, trust in the physician, and ultimately patient adherence [17-20].

While there has been substantial inquiry into the patients information needs in the referral process and methods to address those in other countries, less has occurred in the US [21,22]. Patients are a rich source of information about the health care system and can provide critical data for improving quality [23].

The purpose of this study was to describe the patient's information experience as they managed the transition from their referring physician for the first visit to a specialist physician for a particular condition. The research was part of a larger study of information needs of patients and providers for a quality referral. From prior qualitative work, we hypothesized that there would be a relationship between the quality of the preparatory information that patients received about a first visit to a specialist and their trust in both the referring and specialist physicians. In this study we examined the information that patients received at each point in the referral process, their trust in the referring and specialist physicians, and the role of information in that trust.

## Methods

We used a cross-sectional design to examine the specialist referral experience of individuals diagnosed with a chronic medical condition that resulted in a referral to a specialist. We examined the information that patients received at each point in the referral process, their trust in the referring and specialist physicians, and the impact of the information they received on their trust in the referring and specialist physicians. The study received approval from the University of Kentucky Institutional Review Board for protection of participants.

### Sample

A random sample of 250 patients selected from the National Research Corporation's (NRC) current hospital clients comprised the sample. NRC is a healthcare survey research firm specializing in quality improvement. The patients were selected from 50 hospitals in 45 states, geographically representative, from NRC client base of 500 US hospitals. The sample was considered representative for the population of US patients with a chronic condition, recently (within 6 months prior to the interview) referred to a specialist at any of the National Research Corporation client base hospitals. The patients were contacted by telephone by trained interviewers at the National Research Corporation and informed that the survey was to learn about the experiences people have when their regular family doctor refers them to another doctor for a specific illness or condition. The interview included additional screening to ensure that the individual had a condition that resulted in a referral to a specialist. They were included if they reported they were (1) being treated by a doctor for an ongoing illness or condition, (2) had been seen within the last six months, and (3) were referred by their regular doctor or another doctor. Children and those adults who referred themselves to a specialist were excluded. After accounting for eligibility, the survey process resulted in 250 complete interviews, 7 partial interviews, and 1,275 refusals.

### Survey

The survey used in the research was grounded in the experiences of patients referred by a primary care physician to a specialist. To learn patients' perspective of the referral experience, we first performed a qualitative study of patients with one of five chronic diseases, (congestive heart failure, diabetes, COPD, colon cancer, and breast cancer) that required referral to a specialist provider. Four doctoral-prepared nurses conducted semi-structured interviews with a convenience sample of 50 patients about their experience of being referred for a specialist consultation. The items for the interviews were developed by the principal investigator from review of the literature on patient's evaluation of their health care experience and in collaboration with experts in health care quality. The survey was refined during the interviews by the nurse interviewers and the principal investigator. Respondents were asked to relate the information they were given by their referring physician before their first visit to the specialist, the information they received from the specialist at the visit, their understanding of the information, and what else they would have wanted. Patients expressed a need for preparatory information about their diagnosis, the reason for visit, logistics of getting to specialist, and what to expect at the visit. They also wanted written information and information that was non technical.

The major themes from the interviews were validated in a focus group of patients who had participated in the qualitative study. The themes that emerged were then used to generate items measuring the quality of the information exchange in the referral process. The initial version of the instrument contained 37 items; 16 structured items and two open ended questions concerning information received from the referring physician and 17 structured items and two open ended items about the information that the specialist gave the patient. The questions about information from the primary care provider and the specialist also contained items about the patient's perception of the communication between the referring physician and the specialist. The structured items used a 5 point Likert-type scale (5 = Excellent and 1 = Poor). Feedback from the patient focus groups and the expert evaluations provided support that the domains of the specialist and referring physician's communication quality were well sampled. The item content validity as well as the whole set of items fit the concept and the construct being measured.

For the initial psychometric evaluation, we administered the 37-item survey instrument to a random sample of 231 patients referred to a specialist at an academic health center during a six month period. The response categories were designed to capture the respondent's ratings of quality of the information they received, i.e., excellent, good, fair, poor. The patients often did not receive any information, resulting in many missing values in the data set. The lack of information was as informative as the rated quality of the existing information, thus we added a response category to capture that. Following statistical analysis and use of psychometric criteria for item analysis and reduction [24], the final scale contained a refined set of 21 items, scored on a qualitative scale (5 = Excellent, 4 = Very Good, 3 = Good, 2 = Fair, 1 = Poor, 0 = None/No Information provided).

To assure generalizability to a broader population, the survey instrument was tested with a sample of 250 patients referred to a specialist by a doctor, supplied by the National Research Center . The sample size (n = 250) and number of items (21 items) were adequate to obtain satisfactory level of reliability (at least 10 subjects per item) [25].

### Statistical Analyses

The data were analyzed with the statistical package SPSS 11.5 using descriptive statistics, correlations, factor analysis, and non-parametric tests for independent samples. We compared different aspects of patient's satisfaction among subgroups of patients using Kruskal-Wallis test for independent samples (KWT). The strength of the relationship between any two variables was measured by the Spearman's rank correlation coefficient (Rho) or the Pearson correlation coefficient r and reported by the corresponding p-values (p) for the null hypothesis of no association. Logistic regression modelling was used to test the hypothesis that preparatory information from referring physician is associated with the trust in the physician. We created a new outcome variable "Trust in physician" by dichotomizing the response to the question, *Please, rate how well you trust your referring doctor to coordinate your care about this particular condition*. The response categories Excellent/Very Good/Good were recoded as "Trusted him/her" and the remainder of the response categories were recoded as "Didn't really trust him/her". We assessed the relation between the Patient Centered Referral Information Measure (factors PCPI, PCISP, and PCISM) and the probability that patients trusted the referring physician to coordinate care for the particular condition while controlling for age, race group, income, education, and gender.

Similar analysis was conducted on the self-reported trust in the specialist. A logistic regression model was fit to assess the association between the trust in the specialist and the quality of the information transfer (PCPI, PCISP, PCISM), while controlling for age, gender, race, income, and education level. The dependent variable "Trust in the specialist" was created by dichotomizing the responses to the question *Please, rate how well you trust the specialist you saw*. The response categories Excellent/Very Good/Good were recoded as "Trusted him/her"; the remaining response categories were recoded as "Didn't really trust him/her".

### Psychometric evaluation

The psychometric properties of the instrument were assessed by evaluating construct validity, reliability or internal consistency, temporal stability and predictive validity. The data were subjected to Exploratory Factor Analysis using a Principal Axis Factoring method to extract the initial factors (dimensions) of the measured construct. Using the Kaiser-Guttman rule for eigenvalues greater than 1.0, the initial factor analysis resulted in three factors with eigenvalues of 8.7, 2.7 and 1.5 that together explained 54.9% of the total variance. In order to provide a meaningful interpretation we performed factor rotation. The dimensions of the scale were expected to be correlated and the Oblique rotation was conceptually the most appropriate.

The internal consistency of the items comprising each factor was measured by Cronbach's coefficient alpha. Test-retest reliability was measured before some of the final changes in the wording of the items. Therefore the test-retest reliability was measured for a version of the instrument that underwent slight final changes. However, the non-refined version of the instrument gave very good results for temporary stability. Fifty one patients were interviewed twice within a two month period.

Predictive validity is measured by the size of the correlation score between the scale predictor and a criterion variable [25]. Whether the patients would continue seeing the same specialist and the same referring physician for medical care was hypothesized as an important criterion for the patient's satisfaction with the patient centered information from the two physicians.

## Results

### Sample Demographics

The mean age of the sample was 53 years, 68% were women, 79% were white, and 68% were married. Over half (55%) had some college education and over two-thirds had a total family income before taxes under $40,000. Nearly all participants had some form of insurance and only 6% were uninsured. More than half of the patients rated their general health as at least good (Table 1). The most common conditions were heart disease 8%, back pain 8%, cancer 5%, and lung disease 3%. One half of the patients cited numerous different conditions and 21% did not provide the condition.

**Table 1:** Demographic characteristics of the sample (N = 250)

**Characteristics**	
Gender	
Female	68%
Male	32%
Education	
Never attended school	3%
Grade 1 through 8	4%
Grade 9 through 11	5%
Grade 12 or GED	33%
College 1 to 3 years	38%
College 4 years or more	17%
Ethnicity	
Black/AA	8%
White	79%
Hispanic	4%
Native American	4%
Asian/Pacific Islander	2%
Multiracial	1%
Other	2%
Health Insurance	
Your employer	23%
Someone else's employer	19%
A plan that you or someone else buys on your or their own	5%
Medicare	25%
Medicaid or Medicaid assistance	13%
The military, Champus, Tricare or the VA	5%
Some other source	4%
No insurance coverage	6%
Total family income before taxes	
Less than $10,000	12%
Between $10,000 up to $20,000	14%
Between $20,000 up to $30,000	20%
Between $30,000 up to $40,000	16%
Between $40,000 up to $50,000	6%
Between $50,000 up to $60,000	5%
Between $60,000 up to $70,000	5%
Between $70,000 up to $80,000	1%
Between $80,000 up to $90,000	1%
Between $90,000 up to $100,000	1%
$100,000 and more	2%
Don't know/Refused	17%

### Factor analysis

Analysis of the data revealed that each item was significantly correlated (r ≥ .30) with at least three of the other items, indicating shared common variance. The strength of the relationships ranged from weak (r < 0.29) to strong (0.70 < r < 0.80). All correlations were positive, indicating direct relationships among the items on the scale. The proportion of variance that two items shared ranged from R^2 ^= 0.02 to R^2 ^= 0.54. No inter-item correlation exceeded r = 0.73, indicating no multicollinearity problems. The Barlett's test of sphericity, testing the null hypothesis that there was no linear association among the items, was significant (p-value < .001, χ^2^_df = 210 _= 3081.7). The Kaiser-Meyer-Olkin (KMO) test measuring sampling adequacy produced a KMO measure of 0.92, indicating a high degree of common variance among the 21 items and that if a factor analysis was conducted, the extracted factors would account for a substantial amount of variance. Individual measures of sampling adequacy (MSA) produced very good results, (from 0.86 to 0.95).

Evaluation of the loadings in the factor structure matrix found no items with weak loadings. Two items had strong loadings with two factors. Those items were placed with the factors that were conceptually more closely related. The distinct pattern of item-to-factor correlations and the researchers' hypothesis about the structure of the measured construct led to the decision to keep three distinct factors (Table 2):

**Table 2:** Descriptive Statistics for the Scale Items and Factor Structure Matrix

**Items**	Descriptive Statistics* (n = 250)		**Factor Structure Matrix (Oblique Rotation Promax)**		
			
			**Factors**		
	**Mean**	**StDev**	**1**	**2**	**3**
1. How would you rate the explanation your referring doctor gave you about the reason for seeing a specialist?	4.1	1.2		.60	
2. How would you rate the way your referring doctor explained your condition and prognosis?	3.8	1.4		.64	
3. How would you rate the explanation your referring doctor gave you about the follow-up after the specialist visit?	3.1	1.8			.64
4. How would you rate the written information your referring doctor gave you about your diagnosis?	2.8	2.0			.80
5. How would you rate the written information your referring doctor gave you about your treatment?	2.7	2.0			.79
6. How would you rate the way your referring doctor involved you in making the decision to see a specialist?	3.9	1.5		.79	
7. How would you rate the information your referring doctor gave you about what to expect at the specialist visit, that is, one physician or more, additional tests, etc?	3.2	1.7		.60	
8. How would you rate the way that you referring doctor listened to what you had to say?	3.9	1.4		.79	
9. How would you rate the amount of information you received from your referring doctor?	3.6	1.4		.79	
10. How would you rate the quality of the written information about the appointment, i.e., appointment care, map and directions to the specialist office?	3.4	1.8			.55
11. How would you rate how well your referring doctor prepared you to know what to tell or ask the specialist?	3.1	1.8		.63	
12. How would you rate the information the specialist had about you when you arrived?	3.8	1.5		.55	
13. How would you rate the information the specialist gave you about your diagnosis and your prognosis at your visit?	3.9	1.3	.73		
14. How would you rate the way that the specialist listened to what you had to say?	4.1	1.2	.82		
15. How would you rate the way the specialist involved you in the decisions about treatment?	3.9	1.3	.77		
16. How would you rate the compassion shown by the specialist?	3.9	1.3	.82		
17. How would you rate the information the specialist gave you about follow up care with your primary care provider and or referring doctor?	3.3	1.7			.63
18. How would you rate the information the specialist gave you about what to do if your problems or symptoms got worse?	3.9	1.4	.76		
19. How would you rate the written information (except prescriptions) you got from the specialist about your diagnosis and treatment.	3.5	1.6	.64		
20. How would you rate the amount of time the specialist spent with you?	3.8	1.3	.82		
21. How would you rate the amount of information the specialist gave you about your condition and treatment.	4.0	1.1	.89		

*5 = excellent, 4 = very good, 3 = good, 2 = fair, 1 = poor, 0 = no information

• Factor 1: patient centered information from the specialist physician (PCISP);

• Factor 2: patient centered preparatory information (PCPI) from the referring physician;

• Factor 3: patient centered information given to the patient about self-management (PCISM).

The first two factors, patient centered information from the specialist and preparatory information from the referring were expected from the qualitative stage of the instrument development, but the third factor, information about self-management emerged as a result of the factor analysis and provided new insight and direction for future research.

The common factor analysis produced a rotated factor structure, close to that expected at the development phase of the instrument supporting the construct validity of the instrument. The factor scores were calculated as averages of the scores on all items loading on the corresponding factor. The Alpha coefficient for the factor PCPI was 0.85; for factor PCISP - 0.92; and for factor PCSM - 0.81. The alpha coefficient for the total scale was 0.92. The test-retest correlation for the whole scale was 0.83 indicating good reliability. Although the final version did not undergo test-retest, the changes to the instruments likely would produce the same or better results than the initial version because of the improvements.

One hundred and eighty one patients were still seeing the same specialist while 11 patients had switched to another specialist for this medical problem. The correlation between continuing to see the specialist and PCISP (Patient Centered Information from the SP) was significant (Spearman's rho = .30, p-values < .001) indicating the importance of PCISP in the patient-specialist relationship. A total of 197 patients were seeing the same referring physician while 22 patients were seeing another primary care provider. Patient's perception of the PCPI (preparatory information from the PCP) was significantly correlated with their intent to remain with that physician (Spearman's rho = .26, p-values < .001).

Patients who perceived the information from their referring physicians as more patient centered had greater trust in them (r = .68, p-value < .001). Likewise, patients receiving information from the specialist that was more patient centered were more likely to trust them (r = .77, p-value < .001). There also was a direct relationship between patient's satisfaction with the information received for self-management (PCSM) and the trust in the referring doctor (r = .56, p-value < .001).

Psychometric evaluation of the instrument indicated that the Patient Centered Referral Information Measure (PCRIM) provides a valid and reliable measure of the three components of the quality of patient centered information in the referral process: patient centered information from the referring physician, from the specialist physician, and information about self-management of coordination of care.

### Preparation for the specialist visit

While 85% of patients rated the information the referring physician gave about the referral good to excellent, 8% received no information. Most (90%) thought the referring physician gave a good-to-excellent explanation about the reason for the referral (Table 3). Fewer (80%) expressed satisfaction with the oral explanations about their diagnosis and prognosis. However, over one-fourth of patients received no written information from the referring physician about their diagnosis (26%) or treatment (29%). Of those receiving written information, 16% found the quality of information about their diagnosis and treatment lacking. In contrast, more judged the written instruction about the time and location of the appointment as adequate, yet 15% got nothing in writing. Overall patients thought the amount of preparatory information they received was adequate, but the specific information they needed was lacking.

**Table 3:** Patient's ratings of the quality of information received from the referring physician

**Question**	Ratings of patients who received information*					**Patients who didn't receive any information**
		
	**Excellent**	**Very good**	**Good**	**Fair**	**Poor**	
	**N (%)**	**N (%)**	**N (%)**	**N (%)**	**N (%)**	**N**
How would you rate the explanation your referring doctor gave you about the reason for seeing a specialist?	140(57)	49(20)	37(15)	9(4)	9(4)	6
How would you rate the way your referring doctor explained your condition and prognosis?	114(48)	57(24)	44(19)	12(5)	9(4)	14
How would you rate the explanation your referring doctor gave you about the follow-up after the specialist visit?	84(41)	36(17)	59(29)	12(6)	15(7)	44
How would you rate the written information your referring doctor gave you about your diagnosis?	43(80)	37(20)	38(21)	11(6)	18(10)	66
How would you rate the written information your referring doctor gave you about your treatment?	77(43)	32(18)	46(26)	9(5)	14(8)	72
How would you rate the way your referring doctor involved you in making the decision to see a specialist?	127(54)	43(18)	45(19)	10(4)	10(4)	15(a)
How would you rate the information your referring doctor gave you about what to expect at the specialist visit?	79(37)	49(23)	57(26)	18(8)	13(6)	34
How would you rate the way that you referring doctor listened to what you had to say?	120(50)	51(21)	39(16)	18(7)	15(6)	7 (b)
How would you rate the amount of information you received from your referring doctor?	94(39)	55(23)	56(24)	17(7)	16(7)	12
How would you rate the quality of the written information about the appointment, i.e., appointment care, map and directions to the specialist office?	108(50)	33(16)	50(23)	10(5)	13(6)	36
How would you rate how well your referring doctor prepared you to know what to tell or ask the specialist?	78(37)	48(23)	44(21)	14(7)	26(12)	40 (c)
Overall, how would you rate the information your referring doctor gave you about the referral?	98(43)	51(22)	46(20)	21(9)	14(6)	20
Please rate how well you trust your referring doctor to coordinate your care about this particular condition	141(60)	38(16)	24(10)	17(7)	16(7)	14 (d)

* Percentages are calculated based on the number of patients who received information related to the question -- (a) Didn't involve me at all (b) Didn't listen at all (c) Didn't prepare me at all (d) Don't trust him/her at all

Most patients felt the referring physician listened to them and involved them in decision making. However, more than one in four (26%) felt ill prepared about what would happen at the specialist visit and one-fourth reported feeling unprepared about what to ask or tell the specialist. The referring physician sent information to the specialist for about 50% of patients, but nearly one-fourth of them had to take some or all of the information with them (23%). More than one fourth (27%) reported that their referring physician didn't send anything to the specialist office, didn't give them anything to take, or didn't tell them to get anything for the specialist visit. These patients were significantly less satisfied with how the referring physician prepared them for the specialist visit, their experience with the specialist, and their self-management information when compared with the patients who reported that their physician either sent information to the specialist office or told them to bring the information themselves (KWT p < 0.05). Patients who received all the information needed from the referring doctor (that is test reports, notes, x-rays) to take to the specialist had significantly higher satisfaction with the quality of the information for self-management compared to patients who received no information from the referring doctor for the specialist (KWT p < 0.05).

Most patients continued to see their referring physician after the specialist visit, but 9% found another, and 12% were managed solely by the specialist. Patient's satisfaction with the preparatory information from the referring physician (PCPI) was significantly related to their decision to continue seeing the referring physician versus seeing another primary care physician (logistic regression, p-value < 0.05).

### Patient's experiences at the specialist visit

Most patients felt that specialists were prepared for their visit, though 7% of patients said the specialists had no information about them when they arrived. This is contrary to the 27% that reporting that the referring physician did not send information or ask the patient to take information to the specialist and may be because the referring physician failed to inform the patient that information would be sent. (Table 4 summarizes the results for the specialist). Overall, patients were satisfied with the amount of time the specialist spent with them, although nearly one in five (17%) disagreed. Most patients felt the specialist listened to them, involved them in decision making about their care, and treated them with compassion.

**Table 4:** Patient rating of quality of information about the referral received from the specialist physician

**Question.**	Received Information*					
		
	**Excellent** **N (%)**	**Very Good** **N (%)**	**Good** **N (%)**	**Fair** **N (%)**	**Poor** **N (%)**	**Didn't Receive Information** **N**
**How would you rate....**						

The information the specialist had about you when you arrived?	119(51)	50(21)	43(19)	11(5)	10(4)	17
The information the specialist gave you about your diagnosis and your prognosis at your visit?	120(50)	56(23)	40(16)	14(6)	12(5)	8
The way that the specialist listened to what you had to say?	132(53)	51(21)	40(16)	12(5)	12(5)	3 (b)
The way the specialist involved you in the decisions about treatment?	119(49)	59(24)	38(16)	12(5)	14(6)	8 (e)
The compassion shown by the specialist?	118(48)	58(23)	38(15)	17(7)	16(7)	3
The information the specialist gave you about follow up care with your primary care provider and or referring doctor?	92(43)	46(21)	50(23)	13(6)	14(6)	35
The information the specialist gave you about what to do if your problems or symptoms got worse?	125(53)	57(24)	32(14)	12(5)	10(4)	14
The written information (except prescriptions) you got from the specialist about your diagnosis and treatment?	98(43)	52(23)	51(23)	10(4)	15(7)	24
The amount of time the specialist spent with you?	115(46)	47(19)	45(18)	17(7)	26(10)	NA
The amount of information the specialist gave you about your condition and treatment?	115(46)	67(27)	44(18)	11(4)	11(4)	2
Overall, the way the referring doctor and the specialist coordinated your care?	110(49)	49(22)	39(17)	10(4)	17(8)	25 (f)
How well you trust the specialist you saw?	144(58)	46(19)	29(12)	12(5)	15(6)	4 (d)

* Percentages are calculated based on the number of patients who received information related to the question (b) Didn't listen at all, (d) Don't trust him/her at all, (e) Didn't involve me at all, (f) As far as I know, there hasn't been any coordination at all

Patients gave good ratings to the oral explanations of their diagnosis and prognosis. About 90% got some written materials about their diagnosis and treatment from the specialist, however, not all thought the quality of the written information was good. Most patients (91%) rated the information about what to do if problems or symptoms got worse as good or excellent and only 6% were not told what to do. Thirty percent of patients reported that they had tests repeated that the referring physician had already done. Nearly one in five patients (18%) got information from the specialist that conflicted with what the referring doctor had given.

### Care Coordination

The referring physician and specialist managed the care jointly of 76% of the patients, 12% were solely managed by their referring physician, and 12% were managed solely by a specialist. The majority of patients (80%) trusted the referring doctor to coordinate their care with the specialist. Satisfaction with the coordination of care was highly associated with the quality of information from the referring doctor. Specifically, satisfaction with the way the referring doctor and specialist coordinated the patient's care was significantly associated with their satisfaction with the patient centered preparatory information from the referring physician (PCPI) (Rho = 0.6, p < 0.05), with patient centered information from the specialist physician (PCISP) (Rho = 0.6, p < 0.05), and with patient centered information given to the patient about self-management (PCISM) (Rho = 0.5, p < 0.05).

Over one-fourth (26%) of patients received no information from the specialist about follow-up with their referring doctor and 12% of those who were told about follow-up found the information insufficient. Patients found the coordination of their care by the specialist to be lacking and this perception did not differ based on the patient's gender or age. However, patients with higher education tended to be less satisfied with the information received from the referring physician (PCPI) (KWT p < 0.05) and specialist (PCISP) (KWT p < 0.05), and about self-management (PCISM) (KWT p < 0.05).

Patient satisfaction was greater with the coordination of their care by the specialist when the specialist sent information back to their referring physician. Those feeling that the specialist coordinated their care well were more likely to have been informed by the *specialist *about follow-up care with their referring doctor or primary care provider (Rho = 0.5, p < 0.05). The perception that the specialist coordinated their care well also was highly correlated with how well the *referring physician *explained the follow-up after the specialist visit, prepared them for what to expect at the specialist visit and what to ask or tell the specialist (Rho = 0.5, p < 0.05).(Table 5)

**Table 5:** Care coordination and other information related to the referral

**Question.**	
Did your referring doctor gave you information about how often you might see the specialist	
Yes -	72(29%)
No	178(71%)
Did your referring doctor give you information, that is, test reports, notes, x-rays, to take to the specialist? Did he/she....	
Send everything to the specialist office	123(49%)
Send some information, but you also had to go and get some	27(11%)
Give you everything to take to the specialist office	26 (10%
Tell you where to go to get the information that you needed to take to the specialist	6(3%)
Did not send anything; didn't give you anything/didn't tell you to get anything for the specialist visit	68(27%)
Are you still seeing your referring doctor?	
Yes	197(79%)
No, the specialist is managing my care	31(12%)
No, I am seeing another primary care provider	22(9%)
Did the specialist order any tests and procedures that your or referring had already done?	
Yes	76(30%)
No	174(70%)
Did the specialist give you information that conflicted with what your referring doctor gave you about your problem?	
Yes	44(18%)
No	206(82%)
Are you still seeing the specialist for your condition?	
Yes	181(72%)
No, I don't need to see the specialist for this condition	27(11%)
No, my primary care doctor is managing my care	31(12%)
No, I am seeing another specialist for my problem	11(5%)
When you returned to your primary care doctor did he/she give you the same information about your problem as you got from the specialist?	
Got exactly the same information from my regular doctor that I got from the specialist	122(49%)
Got almost the same information	38(15%)
I am not sure/I haven't seen my regular doctor after the specialist visit	43(17%)
Somewhat different from what the specialist told me	9(4%)
Very different from what the specialist told me	5(2%)
My regular doctor didn't have any information about the specialist visit and couldn't discuss the matter with me	33(13%)

### Physician Trust

Nearly two-thirds of patients (72%) expressed high levels of trust in the referring physician to manage their care, but nearly 20% did not. Those who trusted their referring physician to coordinate their care rated the information from the referring physician higher (Rho = 0.6, p < 0.05), considered themselves more involved in decision making (Rho = 0.6, p < 0.05), and felt the referring physician listened to them (Rho = 0.6, p < 0.05). Trust in the referring physician to coordinate their care was highly associated with their satisfaction with preparatory information from that physician (Rho = 0.7, p < 0.05). The higher the satisfaction with the preparatory information (PCPI) from the referring physician, the greater the trust in him or her (Rho = 0.7, p-value < 0.05). There also was a direct association between patient's satisfaction with the preparatory information for self-management (PCISM) and trust in the referring doctor (Rho = 0.5, p-value < 0.05). The higher the patient's satisfaction with the self-management information, the greater the trust placed in the referring physician.

A logistic regression model testing that preparatory information from the referring physician influenced the patient trust in the referring physician to coordinate their care was significant (Wald p < 0.001). The variable income had a high percentage of (17%) missing values that were recoded into a separate category. Two-way interactions were included and a stepwise method for variable selection was used. Interactions were not significant and were dropped from the model. Only two variables in the full model (see Table 6) had a significant main effect - PCPI (Wald Chi-square p < 0.0001) and PCISM (Wald Chi-square p = 0.04). Therefore patient centered preparatory information from the referring physician and patient centered information given to the patient about self-management are strongly associated with the trust in the referring physician to coordinate the care for the particular condition.

**Table 6:** Logistic regression model with dependent variable "Trust in referring physician"

**Variables**	**Odds Ratio**	**95% Confidence Interval**
Factor PCPI ^1^	9.30	[4.19, 20.64]
Factor PCISM ^2^	1.68	[1.02, 2.76]
Factor PCISP ^3^	0.73	[0.45, 1.20]
Race		
Not White vs. White	0.36	[0.10, 1.32]
Age	1.00	[0.96, 1.04]
Income		
Greater than $40,000 vs. Less than $40,000	0.78	[0.21, 2.83]
Income not Reported vs. Less than $40,000	1.86	[0.34, 10.25]
Education		
HS Graduate vs. Less than HS Graduate	0.44	[0.06, 3.38]
Some College vs. Less than HS Graduate	0.56	[0.06, 5.39]
College Graduate vs. Less than HS Graduate	0.61	[0.08, 4.58]
Gender		
Female vs. Male	0.48	[0.15, 1.58]

^1^PCPI = Preparatory Information from the RP

^2 ^PCIM = Patient Centered Information on Self-management

^3^PCISP = Patient Centered Information from the SP

Patients also expressed a high level of trust in the specialist. Level of trust was strongly associated with receiving good information overall, the specialist understanding of why they were referred, information provided by the specialist about their diagnosis and prognosis, and information about how to care for themselves at home (p-values for the Spearman correlation tests were less than 0.0001). Trust in the specialist was also strongly linked to the patient centeredness of the specialist, being listened to, involved in decision making, and treated with compassion (Spearman correlation test p-values < .0001).

The logistic regression model testing the association of the quality of the referral information produced a significant p-value for the full model. Only one independent variable, patient centered information from the SP (PCISP), had a significant main affect (Wald Chi-square p < 0.0001). Thus trust in the specialist was strongly associated with the patient centeredness of the specialist. (Table 7)

**Table 7:** Logistic regression model with dependent variable "Trust in specialist"

**Variables**	**Odds Ratio**	**95% Confidence Interval**
Factor PCISP	8.44	[4.12, 17.27]
Factor PCISM	0.84	[0.47, 1.52
Factor PCPI	1.06	[0.55, 2.05]
Race		
Not White vs. White	3.71	[0.51, 27.09]
Age	0.97	[0.92, 1.03]
Income		
Greater than $40,000 vs Less than $40,000	1.23	[0.25, 6.10]
Income not Reported vs. Less than $40,000	0.52	[0.11, 2.53]
Education		
HS Graduate vs. Less than HS Graduate	0.42	[0.04, 4.15]
Some College vs. Less than HS Graduate	1.99	[0.12, 32.67]
College Graduate vs. Less than HS Graduate	0.32	[0.03, 2.89]
Gender		
Female vs. Male	1.43	[0.42, 4.91]

## Discussion

Our findings illuminate the critical role played by preparatory information in the coordination of care between the primary care provider, the patient, and the specialist provider. Patients were more satisfied if they were well informed. Most patients received good information about the reason for the referral to a specialist, but were not prepared for what to expect at the specialist visit. Most explanations were oral and the written information that patients received was less than adequate. That patients received different information about their condition from the specialist is to be expected because the specialist has more in-depth knowledge about the problem.

The number of repeated tests (30%) reported by patients is higher than prior studies and raises concern about the increased costs in the system resulting from insufficient information exchange between providers [26]. Although some redundant testing is warranted the estimated annual savings resulting from electronic information exchange is over one billion US dollars [27]

This study did not address the perspectives of the referring or specialist physicians, consequently the method of information exchange between the providers is not known. An earlier study by the investigators of 231 regional referrals found that the primary care providers most often used facsimile transmission with only one using email.

The quality of the preparatory and self-management information given to patients by their primary care providers influenced their trust in the ability of the primary care provider to coordinate their care and also influenced their trust in the specialists. Hence the quality of preparatory information given to patients is very important. Knowing the ratings on the items in these scales that predict trust can assist the referring and specialist physicians in identifying weaknesses and plan interventions to improve the process and subsequently increase the trust that patients have in them. Each of these processes could be tractable to improvement; and it should be possible to develop tools to assist the primary physicians in providing good preparatory information.

There are limitations to this study including the use of a random sample of patients selected from the National Research Corporation's (NRC) current hospital clients. The sample is not a random sample of all US patients, only those served by the 500 client hospitals of NRC and the findings can only be generalized to the population of NRC hospitals. That only 1 in 7 agreed to the respond to the survey is also a limitation. Some authors suggest that there is an increasing trend toward refusals particularly in telephone surveys that can be attributed to time constraints ("too busy"), lessened sense of civic responsibility or sense of reciprocity, too many survey requests, and concerns about safety, fraud, and misrepresentation. Low response rates create the possibility for nonresponse bias. It is not known if those who responded to the survey differ from those who did not. We are assuming that the results from the survey are generalizable, but further research will show whether this assumption was correct. The use of a single- item to measure trust in the referring physician to manage the referral process and trust in the specialist also may be considered a limitation. Although single item measures have been reported in other studies of care transitions, and are appropriate for some purposes, multi-item measures are useful for identifying specific areas for improvement [28-30].

## Conclusion

Referrals are very common - in 2006 about 33% of the 421 million specialist's visits resulted from a referral - and constitute an important transition in care from the primary physician to a specialist and then back again. Ideally all or nearly all patients should feel that they got good information about the referral.

It is likely that primary physicians do not get good feedback from patients or specialists about the adequacy of the information they have provided. Primary care physicians are key to improving coordination of care by managing referrals [31]. If physicians are to improve their information exchange with patients, certain changes in the health system could help support the process. The current movement in the US towards use of electronic health records with electronic health information exchange between providers would increase provider to provider communication and allow for patient access to their pertinent information. Perhaps more importantly, the designation of a medical home (primary care provider) for all patients would improve coordination of care. Patients would know who was responsible for their care and the primary care provider would be responsible for coordinating information exchange with other providers in the system.

## Competing interests

The authors declare that they have no competing interests.

## Authors' contributions

CLI participated in the study design, survey development, provided oversight for the survey, participated in data analysis, and drafted the manuscript. CLS participated in the design of the survey instrument and manuscript review. FDS participated in the design of the study and drafting of the manuscript. SS conducted all of the statistical analyses and assisted in drafting the manuscript.

## Authors' Information

CLI has over 40 years in health care with an emphasis on quality measurement, served on a NCQA expert panel on quality metrics in the care transition, and serves on the board of a national QIO.

SS is the Assistant Director for Research and Analysis in the Office of Institutional Research at the University of Kentucky and has published in US and international journals.

CLS is the Chief Medical Information Officer at an academic health center and developed a web based portal for referring physicians.

FDS has been involved in health services education and research for over forty years, has published extensively, served in editorial positions for eight health related publications, and on numerous national organizations.

## Pre-publication history

The pre-publication history for this paper can be accessed here:


